# Social Cognition Capacities as Predictors of Outcome in Mentalization-Based Treatment (MBT)

**DOI:** 10.3389/fpsyt.2020.00691

**Published:** 2020-07-22

**Authors:** Elfrida H. Kvarstein, Espen Folmo, Bjørnar T. Antonsen, Eivind Normann-Eide, Geir Pedersen, Theresa Wilberg

**Affiliations:** ^1^ Section for Personality Psychiatry, Clinic for Mental Health and Addiction, Oslo University Hospital, Oslo, Norway; ^2^ Institute for Clinical Medicine, University of Oslo, Oslo, Norway; ^3^ Department of Psychiatry, Lovisenberg Diaconal Hospital, Oslo, Norway; ^4^ Department of Research and Development, Clinic for Mental Health and Addiction, Oslo University Hospital, Oslo, Norway

**Keywords:** social cognition, personality disorder, mentalization-based treatment, MASC, Borderline personality disorder

## Abstract

**Background:**

Mentalization-based treatment (MBT) is an evidence-based treatment for borderline personality disorder (BPD). Differences in treatment outcomes related to specific capacity of social cognition need further attention. This study aimed to investigate social cognition as a predictor of outcome.

**Method:**

The study included 31 BPD patients who completed a test of social cognition (Movie for the Assessment of Social Cognition, MASC) before outpatient MBT. The MASC-scores indicated a person’s theory of mind (ToM) and different error-types. During treatment repeated self-reports of alliance and clinical outcomes (symptoms, interpersonal problems, social functioning) were applied. Longitudinal analyses were based on Linear Mixed Models (n = 24).

**Results:**

The most frequent error-type was excessive ToM (hypermentalizing). Higher levels of excessive ToM were associated with greater improvement of alliance over time and good clinical outcomes. Insufficient ToM errors and low levels of accurate cognitive ToM responses were both associated with poorer improvement over time. The subgroup with frequent insufficient ToM errors had a larger total number of ToM errors. Insufficient ToM errors were associated with more childhood trauma, comorbid avoidant PD traits and/or PTSD, extensive prior treatment, and/or treatment irregularity.

**Conclusion:**

This study demonstrates considerable variation of social cognitive capacity among BPD patients and good outcomes for patients with mainly ToM errors of hypermentalizing. It also indicates that poorly responding patients may represent a cohort with more complex problems of social cognition and insufficient mentalizing.

## Introduction

The concept of mentalization has in later years been particularly advanced in treatment of patients with borderline personality disorder (BPD) (1). It refers to the human ability to perceive and interpret emotional and intentional states of mind, and includes awareness of own mental state (self-awareness, self-reflection) and sensitivity to others, also termed social cognition or theory of mind (ToM) (2). The present study focuses on social cognition among patients with BPD. It explores if and how impairments of such interpersonal understanding may predict the long-term course of treatment.

Within clinical BPD samples, it is reasonable to assume that mentalizing capacities may vary as the clinical presentation of BPD and its severity is known to be heterogenous (3), Central BPD features are emotional dysregulation, impulsivity, and unstable relationships (1) and severe situations such as suicide attempts and acts of self-harm are often linked to emotional dysregulation (4). Comorbidity is common within BPD samples and avoidant PD has more recently been highlighted as a complicating condition (5, 6). Interpersonal vulnerability, emotional dysregulation, and comorbidity of other personality disorder traits have all been associated with impaired mentalizing (7–10). However, few studies have specifically investigated the relational aspect of mentalizing—social cognition.

Studies of mentalizing use different evaluation methods. In several studies, mentalizing capacity, conceptualized as reflective functioning (RF), has been evaluated on the basis of the Adult Attachment Interview (11–13). Other, related developments include RF assessments based on transcripts of therapy sessions (14), and also patient’s self-report (15). Differing from both observational assessment and self-report, the Movie for the Assessment of Social Cognition (MASC) invites the test participant to actively, interpret ongoing social interactions shown on the movie (16). As a test of current theory of mind, the MASC addresses participants’ immediate understanding of other people’s thoughts, intentions, or emotions. The test differentiates between impairment in terms of a lack of ToM, insufficient ToM, or excessive ToM (hypermentalization). Former MASC-based studies of social cognition among BPD patients have indicated a tendency for hypermentalization (8, 17). Nevertheless, although social cognition is a central aspect of psychopathology, the clinical implications of different types of mentalizing problems are still poorly described.

Mentalization-based treatment (MBT) is an evidence-based, long-term treatment program recommended for patients with severe BPD (18, 19). It combines individual and group psychotherapy. The overriding aim is to stabilize and enhance patients’ mentalizing capacity. Keeping a therapeutic focus on mental states, interpersonal interpretation, and interaction, it provides an explicit challenge of mentalizing others, both therapists and group members. MBT studies have indicated that this specialized approach is particularly justified for BPD patients with severe disorder (20–22). For patients, the treatment, interventions, and formats, represent complex social situations and will activate all aspects of social cognition. An essential question is therefore how MBT is able to accommodate BPD patients with more severe impairment of social cognition. As yet, no studies have investigated how clinical outcomes in MBT or other psychotherapies may vary according to the patient’s specific capacity for social cognition.

The current investigation is a small, longitudinal study of poorly functioning BPD patients in regular outpatient MBT. The study primarily aims to investigate pretreatment social cognitive capacity as a predictor of clinical improvement and alliance development over time. In accordance with former research, we expect that problems of hypermentalization will dominate the sample of BPD patients, and we hypothesize that more extensive hypermentalizing problems will be associated with poorer clinical improvement and poorer treatment alliance. In secondary analyses, we aim to further explore variation of pretreatment impairment in social cognition and how such impairment is associated with other aspects of clinical severity—severity of BPD, comorbidity, reported childhood trauma, risk status, and treatment factors.

## Material and Methods

### Design

The study is an observational, longitudinal study.

### The Sample

The sample includes BPD patients treated within an outpatient clinic on a specialist mental health service level. During the period 2012–2014 patients referred to the clinic were invited to participate in a cross sectional study of social cognition (17). A total of 31 patients who had volunteered for this study and completed a test of social cognition (Movie for the Assessment of Social Cognition, MASC) were then, on a regular clinical basis, admitted to treatment in an MBT program. All patients admitted to MBT who had a MASC test were eligible for the present investigation. Description of the sample is presented in **Table 1**.

**Table 1:** Clinical status on referral to MBT.

Total N = 31	Total sample N = 31	
	Mean(SD)	%
Age	27.6 (5.5)	
Gender female		81
*Overall functioning*		
No work/study at all last year		45
Observer-rated GAF	48 (4.9)	
Self-report WSAS	26 (7.0)	
*Problems & symptoms*		
Sum RSES	1.9 (0.6)	
Sum CIP	1.9 (0.5)	
Sum BSI	2.1 (0.8)	
*PD status*		
Borderline PD		90
Borderline PD traits	6.2 (1.5)	
Number PD traits	15.1 (5.9)	
Number of PDs	1.5 (0.8)	
NOS PD		7
*Specific PD comorbidity*		
Schizotypal		0
Paranoid		13
Antisocial		3
Narcissistic		0
Avoidant		10
Obsessive Compulsive		16
Dependent		10
*Symptom disorders*		
Total number	2.2 (1.6)	
Mood		61
Anxiety		58
PTSD		20
ADHD		7
Eating		16
Substance abuse		10
Autism		0
Dissociative		0
Psychosis		0

### The MASC Test of Social Cognition

The primary research aim was to investigate social cognition as a predictor. Social cognition was systematically assessed at baseline, by a researcher (third author, not a therapist) before patients started treatment with the movie-based test, MASC (16), a 15-min movie about four people coming together for a dinner party. MASC test scores were not available for clinicians. The movie is paused at regular intervals, 41 times, and the study participants are given questions concerning the characters’ feelings/emotions, thoughts, and intentions (e.g. “what is Cliff feeling?” “what is Michael thinking?” and “why is Sandra asking this?”). Altogether, the MASC includes 18 questions concerning emotions, 9 questions about thoughts, and 17 about intentions. For each question, the given response options are categorized as accurate or inaccurate. An inaccurate response, suggesting inadequate ToM, is subdivided into excessive, insufficient, and a no ToM response. Excessive ToM reflects assumptions about other people’s mental states, beyond what most other observers would find reasonable. Insufficient ToM reflects mental state inferences that are imprecise or incorrect. No ToM reflects non-mental state inferences, e.g. physical causation (16, 17). An accurate MASC response, suggesting adequate ToM, can again be classified as an accurate perception of thoughts, intentions, or emotions. In this study the MASC test provides eight different scores: The total number of errors, number of insufficient, excessive and no ToM errors, and the total number of accurate interpretations, number of accurate interpretations of thoughts, intentions, and emotions.

### Repeated Assessments of Treatment Alliance and Clinical Outcomes

The primary aim of the study was to investigate social cognition as a predictor of alliance development and clinical outcomes. The longitudinal design of the study implies that assessment of clinical outcomes and alliance data were repeated for each individual over the course of treatment. Mean number of assessments was 2.9 (*SD* = 1.7, range 1–7) over a maximum of 3 years. The study does not include any follow-up investigation after treatment termination. Seven patients had not completed any self-reports. Longitudinal data of clinical outcomes were thus available for 24 patients. All evaluation measures were a part of the treatment unit’s regular assessment procedures, and were applied repeatedly during the whole treatment period. Assessments were not blind to the therapists, but functioned as feed-back and evaluation during treatment.

The repeated evaluation instruments included only standardized, validated instruments:

1. The *Global Assessment of Functioning* (GAF: APA, 1994) an observer-based evaluation of functioning and symptom severity (1–100 scale). Clinicians in the MBT team were trained to evaluate GAF. Reliability has been found acceptable in this context, with a generalizability coefficient of.84, for relative decision and.82 for absolute decisions (23). Clinically relevant impairment is indicated by GAF <60, with lower scores indicating increasingly poorer functioning.
2. The *Work and Social Adjustment Scale* (WSAS) (24), a patient self-report five-item measure of functional impairment (0–8 scale, score 0 indicating no impairment at all, 8 severe impairment, and a sum score ranging from 0–40). The WSAS is a reliable instrument, measuring individual variation in clinically important aspects of impairment (25). Severe impairment is indicated by high scores.
3. *Brief Symptoms Inventory* (BSI-18) (26), a short form of the Revised Symptom Checklist 90 (27). The BSI-18 is a patient self-report which comprises 18 of the items from SCL-90-R and three areas of distress: Somatization, Depression, and Anxiety. All items are rated on a five-point Likert scale (range 0–4, from “not at all” to “extremely”). A total sum-score is computed. Severe distress is indicated by high scores.
4. *Circumplex of Interpersonal Problems* (CIP) (28), a 48-item Norwegian version of the patient self-report Inventory of Interpersonal Problems—Circumplex version (29). CIP has a five-point (0–4) response format (“not at all” to “extremely”). Total mean CIP is reported. Severe interpersonal problems are indicated by high scores.
5. *Rosenberg self-esteem scale* (RSES), a widely used, 10-item self-report inventory assessing positive and negative feelings about him/herself (30). All items are answered using a four-point Likert scale format (from “strongly agree” to “strongly disagree”). Low self-esteem is reflected by a low score.
6. The *Working Alliance Inventory—revised short version* (WAI-SR) (31) is a 12-item self-report questionnaire rated on a Likert scale from “never” (1) to (7). The patients filled out WAI-SR; with reference to their individual therapist (WAI-SR_i_). We report the mean sum-score of WAI-SR_i_.

### Sociodemographic Information

Sociodemographic information was recorded by patient self-report for all patients at baseline and included information about self-harming and suicidal behaviors, episodes of violence, previous psychotic episodes, former treatment experience, and childhood trauma. Treatment duration, treatment regularity, and reasons for treatment termination were recorded by therapists at discharge. This information was based on a questionnaire used within the MBT unit, originally designed for use in a collaborative cross-regional clinical quality and research Network (Network for personality disorders). For use in the secondary analysis, sum-scores were calculated by combining variables for self-harming and suicidal behaviors (suicide/self-harm risk score), violent acts (violence risk score), childhood trauma (sum childhood trauma), and previous treatment (sum prior treatment).

### Assessment of Diagnosis

All patients were diagnosed before starting treatment in accordance with the DSM-IV using the Mini International Neuropsychiatric Interview, (M.I.N.I.) (32) for symptom disorders and the Structured Clinical Interview for DSM-IV Axis II Personality Disorders (SCID II) (33) for personality disorders. Diagnostic assessments were performed by therapists at the MBT unit who had received systematic training in diagnostic interviews and principles of the LEAD-procedure (Longitudinal, Expert, All-Data) (34, 35) where diagnoses were based on all available information together with the two diagnostic interviews. Variables based on SCID II and M.I.N.I. were used in the secondary analyses.

### The Treatment, Therapists, and MBT Fidelity

The MBT program was applied in accordance with guidelines and manuals (36–38), allowing for up to 36 months total treatment duration. In the first year, patients attended 12 sessions in an MBT psychoeducational group and weekly MBT individual and group therapy sessions (1.5 h). Frequencies of individual therapy were gradually reduced, while weekly group sessions continued throughout treatment. The outpatient clinic served an urban population, aimed to treat poorly functioning young adults (18–30 years) with BPD, and had a total MBT capacity of 64 patients. Patients who did not have BPD were not admitted to MBT, nor were patients with unstable bipolar disorder, schizophrenia, or autism spectrum disorder.

The MBT therapists were experienced and had additional MBT training (psychiatric nurses, psychiatrists, psychologists, an art therapist, and a social worker), eight were qualified group analysts, two in psychoanalysis/psychodynamic psychotherapy, 67% were females, and mean age (year 2012) was 56 (*SD* = 9) years. Other therapists in the research period were resident doctors and psychologists in training. MBT therapists met for weekly video-based group supervision by supervisors with MBT training and one-day supervision seminars every half year. The first, second, and fourth author were therapists within the MBT unit in the study period.

The assessment of therapists’ adherence to the MBT model (MBT fidelity) was provided by the MBT quality laboratory—an available cross-regional supervisory service supporting implementation and quality of MBT. The service was allocated within a National Advisory Unit for Personality Disorders. MBT therapists could send in videos of therapy sessions for evaluation and received fidelity scores and personal feed-back. Fidelity was rated by the MBT Adherence and Competence Scale and the Adherence and Competence Scale for Mentalization-based Group Therapy and based on video-recorded therapy sessions. Both rating-scales have been found reliable (39, 40). A group of five raters evaluated 19 individual sessions (eight therapists in the program) and nine group sessions (period 2013–15). All raters had part-time affiliations as therapists in the MBT unit. For the individual therapy, the mean adherence-level was 4.7 (*SD* = 1.2) and mean competence-level 4.4 (*SD* = 1.2), and for group therapy, the mean adherence score was 5.1 (*SD* = 1.4) and mean quality rating (competence) was 4.9 (*SD* = 1.3). According to these measures, “good enough” adherence and competence is defined as level 4 (1–7 scale).

### Ethics

All data were extracted from an anonymous clinical research database with approved procedures and patients’ written, informed consent. The database includes longitudinal data from patients referred to treatment on a regular basis. All clinical data are based on regular evaluation instruments used in the clinic. Patients in this study had for research purposes in a former study, performed a single MASC test. MASC scores were available in the anonymous data set.

### Statistical Procedures

Mixed models (MM) is a methodology based on the principles of maximum likelihood (41) was used in the main statistical analyses (Mixed Models, IBM SPSS statistics version 25). MM was chosen because of a) the longitudinal study design where each individual represents a cluster of dependent data, b) the data from this clinical sample were unbalanced, and c) the long-term data collection. MM methodology is designed for analyses of clustered data (41, 42). MM estimations of change for each individual combine all available observations of the person, and model based estimations do not require that all subjects have equal numbers of assessments or that the time intervals between assessments are constant (43). MM can incorporate heterogenous variation (41). Modeling procedures were stepwise.

#### Step 1: Longitudinal Models of Outcome and Alliance

Step 1 represented the establishment of longitudinal models. The six variables 1) GAF, 2) BSI, 3) WSAS, 4) CIP, 5) RSES, and 6) WAI_i_-SR were dependent variables. Time (months from baseline) was added as a continuous variable in all models. The time-points of repeated assessments were 3 months, 6 months, 12 months, and then every sixth month until 36 months. In addition, dependent variables were assessed at the time of treatment termination. In order to balance data against a possible trend of linear inflation, termination scores were all placed at the last time point. Best model fit was found for models with time as a continuous variable, specification of intercept as a random effect, and unstructured covariance. Linear trajectories captured significant longitudinal trends in the data for all dependent variables (*p* < 0.001), and log likelihood estimations of model fit indicated significant improvements from an unconditional model to the linear random coefficient model (critical values for chi-square statistic: *p* < 0.01). Basic requirements of normally distributed residuals were acceptable.

#### Step 2: Investigation of MASC Scores as Predictors of Outcome and Alliance

Step 2 represented the main predictor analyses. The eight continuous MASC variables were each investigated as predictors in separate models. The MASC variables were investigated by adding the terms MASC (controlling for baseline variation) and the longitudinal term: (MASC * linear time) to the linear step 1 model. For interpretation of results, each predictor model is judged by the associated deviation of the trajectory of the dependent variable after adding the MASC variable. MM estimations in this study included intercept, slope, the corresponding change in intercept variance, the remaining residual variance, and change in estimates of log likelihood statistics (Indices of model fit, Akaikes Information Criterion, AIC). Reduction in variation is presented as % explained variation indicating % change from the estimated variation in the initial step 1 reference model. In figures illustrating differences in longitudinal trends, two dichotomous variables were introduced, one for insufficient ToM errors, and one for excessive ToM errors. The dichotomous variables indicated low score levels (average or below) and high score levels (above average).

#### Secondary Analyses

Secondary analyses aimed for further exploration of factors associated with variation of MASC ToM errors. Statistical comparison of descriptive MASC data were based on independent sample T-tests. Two MASC variables were investigated as dependent variables, a) the total number of MASC errors and b) the total number of insufficient ToM errors. Best model fit was found for Step 1 models with intercept as a fixed effect, no random effects, and unstructured covariance. Secondary analyses included Step 2 models adding as predictors, the dichotomous variable for insufficient ToM errors (model a), and in separate models, comorbid disorders, childhood trauma, risk scores, and treatment factors (model b).

#### Missing Assessments

Patients in the sample had different numbers of assessments (range 1–7). Among the 24 patients who had filled in self-reports, 29% had only two assessments, 50% had at least three assessments, and 17% had five or more. The number of assessment points was added as a categorical predictor in clinical outcome and alliance models in order to investigate possible longitudinal bias of missing assessments (44). The number of assessments was not associated with deviation of change-rates (for all models p_LM_ < 0.05). Among the seven patients who lacked self-reports, mean baseline GAF was 52 (SD 5), and mean treatment duration was 9 months (SD 8, range 2–24). Four patients were early treatment drop-outs (treatment duration, range 2–4 months).

## Results

### Description of Baseline Status at the Start of MBT

Mean age was 28 and the majority were females. All patients were diagnosed with BPD. The majority had comorbidity of other PD traits, PDs, and symptom disorders. Comorbid PTSD was present in 20%. Observer-rated scores of GAF, self-reported WSAS, BSI, and reports on work/study activity last 12 months indicated poor psychosocial functioning and high levels of distress, and RSES and CIP revealed poor self-esteem and relational problems (**Table 1**).

### Step 1: Longitudinal Models of Outcome and Alliance

A similar longitudinal trend of clinical improvement was found for all variables: 1) GAF, 2) WSAS, 3) BSI, 4) CIP, and 5) RSES (for all dependent outcome variables: *p*_MM slope_ < 0.001), together with increasing levels of WAI-SR_i_ (*p*_MM slope_ < 0.05). The within-subject effect size (paired samples, repeated measures cohens *d* = (*m*1−*m*2)/√*sd* ^2^_1_+*sd* ^2^_2_− (2*rs*_1_ *s*_2_)) for GAF was 0.8. However, significant residual variation was evident for the clinical outcome and alliance measures (for all: *p*_MM residual variation_ <0.001). This result suggests further investigation of possible predictors. Linear Mixed Model estimations are shown in **Table 2**.

**Table 2:** Outcomes, alliance, and MASC ToM scores—mixed model estimations.

LMM		Intercept estimate	Linear slope estimate	Explained intercept variation	Explained residual variation	Model fit
Model	Predictor	Mean (SE)	Mean(SE)	%	%	AIC
**1) GAF**		49 (0.9)	0.3 (0.06)***	*Ref. ns.*	Ref. 28 (5)***	586
	Insufficient	ns	−0.05 (0.03)*		14	579
	Excessive	ns	ns		4	586
**2) WSAS**		25.1 (1.4)	−0.21 (0.06)***	Ref. 24 (11)*	Ref. 59 (10)***	731
	Insufficient	ns	0.07 (0.02)**	4	9	723
	Excessive	ns	ns	8	2	732
	Thoughts	ns	−0.18 (0.07)**	4	9	728
**3) BSI**		2.1 (0.13)	−0.02 (0.005)***	Ref. 0.23 (0.09)*	Ref. 0.48 (0.07)***	278
	Insufficient	ns	0.01 (0.002)**	4	8	272
	Excessive	ns	ns	4	0	280
	Thoughts	ns	−0.01 (0.005)*	0	4	277
**4) CIP**		1.9 (0.08)	−0.01 (0.003)***	Ref. 0.096 (0.04)**	Ref. 0.17 (0.03)***	162
	Insufficient	−0.08 (0.03)*	0.004 (0.001)***	0	12	156
	Excessive	ns	−0.002 (0.001)*	0	6	162
**5) RSES**		1.84 (0.09)	0.02 (0.004)***	Ref. 0.11 (0.05)*	Ref. 0.25 (0.04)***	181
	Insufficient	ns	−0.004 (0.002)**	0	8	177
	Excessive	ns	ns	0	0	182
**6) WAI-_I_ SR**		4.86 (0.24)	0.02 (0.01)*	Ref. 0.81 (0.11)*	Ref. 0.532 (0.3)***	201
	Insufficient	ns	ns	0	2	203
	Excessive	ns	0.005 (0.002)*	7	6	200

**Table 2** demonstrates linear mixed model estimations for the six dependent variables, baseline (intercept estimates and % explained variation) and longitudinal deviation (slope estimates and % explained variation) associated with MASC scores indicating “insufficient” and “excessive” theory of mind errors and accurate responses for the cognitive subdomain, “thoughts.” Significant differences are marked with * (p < 0.05), **(p < 0.01), or ***(p < 0.001). Nonsignificant differences are indicated by ns. Indicator of model fit is Akaikes Information Criterion (AIC). Lower value indicates better model fit. The first row for each investigated dependent variable indicates Step 1 analyses. The following rows for each dependent variable indicate Step 2 analyses—one row for each separately investigated ToM predictor.

### Step 2: Main Analyses, MASC Scores as Predictors of Outcome and Alliance

**Figure 1:**
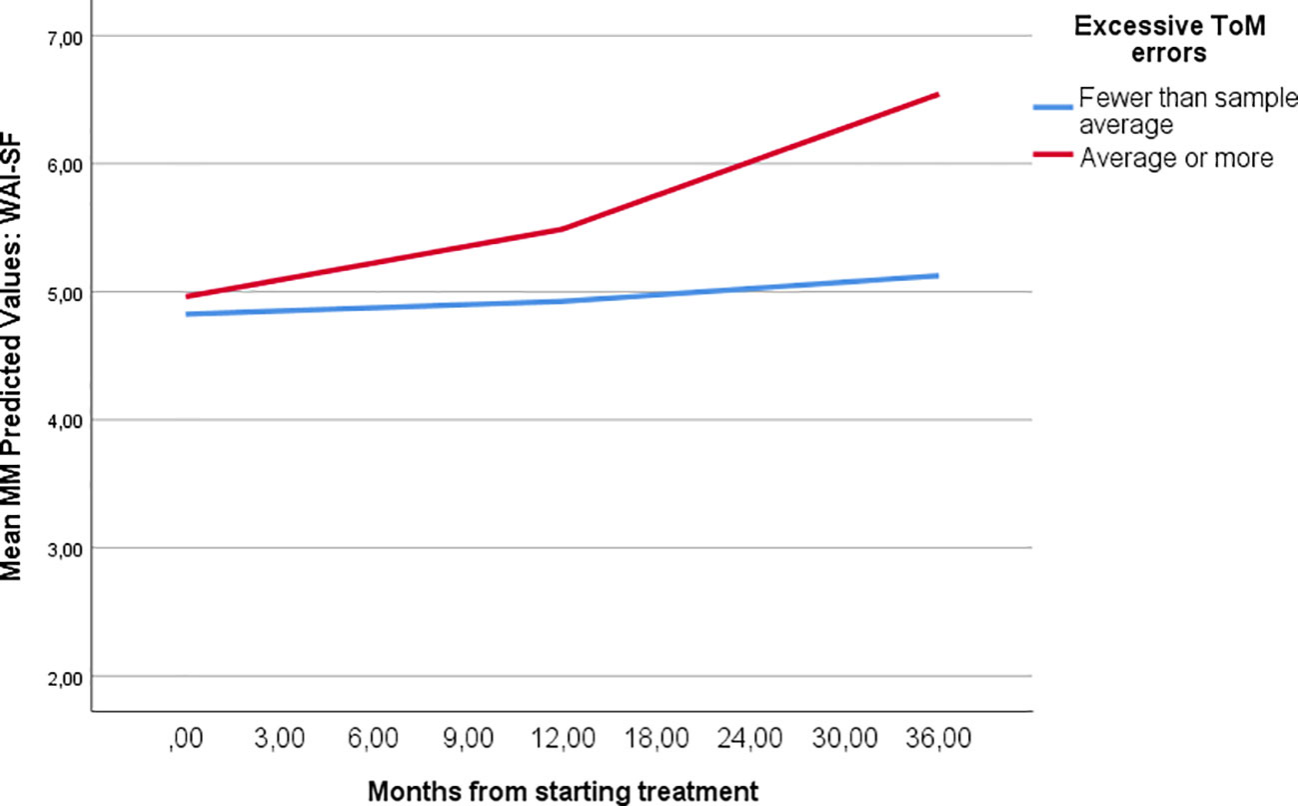
Therapeutic alliance development during MBT relative to ToM error patterns. **Figure 1** demonstrates the different course of treatment alliance among patients with higher and lower levels of excessive ToM errors according to the MASC test. Trajectories are based on MM estimations.

Excessive ToM errors were associated with enhanced improvement-rates of variable 4) CIP (*p*_MM slope_ < 0.05). Excessive ToM errors did not predict deviating change for the other outcome variables; 1, 2, 3, and 5 (*p*_MM slope_ > 0.05). Across the five outcome models, excessive ToM errors explained a mean of 2.4% (range 0–6) residual variation, but did not improve model fit. Excessive ToM errors were significantly associated with enhanced positive development of WAI-SR_i_ (*p*_MM slope_ < 0.05), explained 6% residual variation and improved model fit. Excessive ToM errors were not associated with deviating baseline levels of clinical outcome measures and initial alliance (*p*_MM intercept_ > 0.05). Estimations are shown in **Table 2** and **Figure 1**.

**Figure 2:**
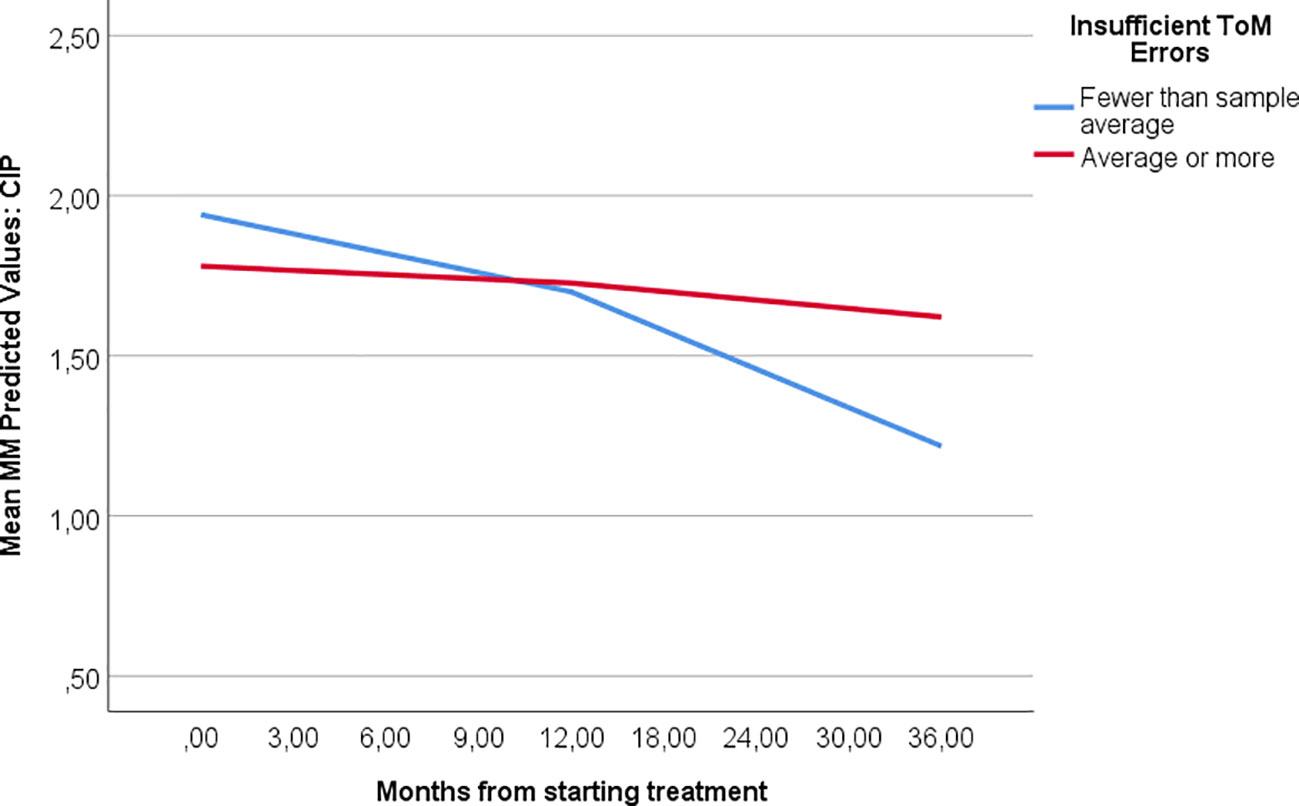
Outcomes in MBT with different ToM error patterns. **Figure 2** demonstrates the different course of treatment among patients with higher and lower levels of insufficient ToM errors according to the MASC test. Trajectories are based on MM estimations.

Insufficient ToM errors were strongly associated with poorer rates of clinical improvement across all five outcome variables (*p*_MM slope_ < 0.05). In the five outcome models, insufficient ToM errors explained a mean of 10.2% (range 8–14) residual variation, and improvement of model fit was apparent in all five models. Insufficient ToM errors did not predict deviating development of WAI-SR_i_ over time (*p*_MM slope_ > 0.05), explained 2% residual variation, but did not improve model fit. Insufficient ToM errors were not associated with deviating baseline levels of clinical outcome measures and initial alliance (*p*_MM intercept_ > 0.05). Estimations are demonstrated in **Table 2** and **Figure 2**.

Accurate interpretations of thoughts were associated with enhanced improvement of two of the five outcome variables (2) WSAS and 3) BSI), explaining 4 and 9% residual variation, and improving model fit, (*p*_MM slope_ < 0.05). Accurate interpretations of thoughts did not predict deviating change of the remaining three outcome variables or alliance development (*p*_MM slope_ > 0.05). Across the five outcome measures, the number of accurate interpretations of thoughts explained a mean of 2.6% outcome variation (range 0–9%) and alliance development 0%. The predictor was not associated with deviating baseline levels of clinical outcome measures and initial alliance (*p*_MM intercept_ > 0.05). Estimations are demonstrates in **Table 2**.

The total number of ToM errors and the total number of accurate ToM responses did not predict any clinical outcomes or alliance development (*p*_MM slope_ > 0.05). In the five outcome models, these predictors explained respectively, a mean of 0.6 and 0.8% residual variation (range 0–2 and 0–4%). In the alliance model, accurate ToM responses did not explain residual variation, but the total number of ToM errors explained 1%. None of the predictors improved model fit. The predictor was not associated with deviating baseline levels of clinical outcome measures and initial alliance (*p*_MM intercept_ > 0.05).

The variable no ToM errors did not predict deviating change of any of the outcome variables (*p*_MM slope_ > 0.05). In the five outcome models, No ToM errors explained a mean of 1.1% (range 0–6) residual variation, and did not improve model fit. No ToM errors did not predict deviating development of WAI-SR_i_ (*p*_MM slope_ > 0.05), but explained 2% residual variation and improved model fit. No ToM errors were not associated with deviating baseline levels of clinical outcome measures and initial alliance (*p*_MM intercept_ > 0.05).

Accurate interpretations of intentions and emotions did not predict outcome deviation for any of the clinical outcome variables (all *p*_MM slope_ > 0.05). Accurate interpretations of intentions and emotions explained each a mean of 0.8% (for both: range 0–4%) residual variation across all five clinical outcome measures. The predictors were not associated with deviating baseline levels of clinical outcome measures and initial alliance (*p*_MM intercept_ > 0.05).

### Secondary Analyses

**Figure 3:**
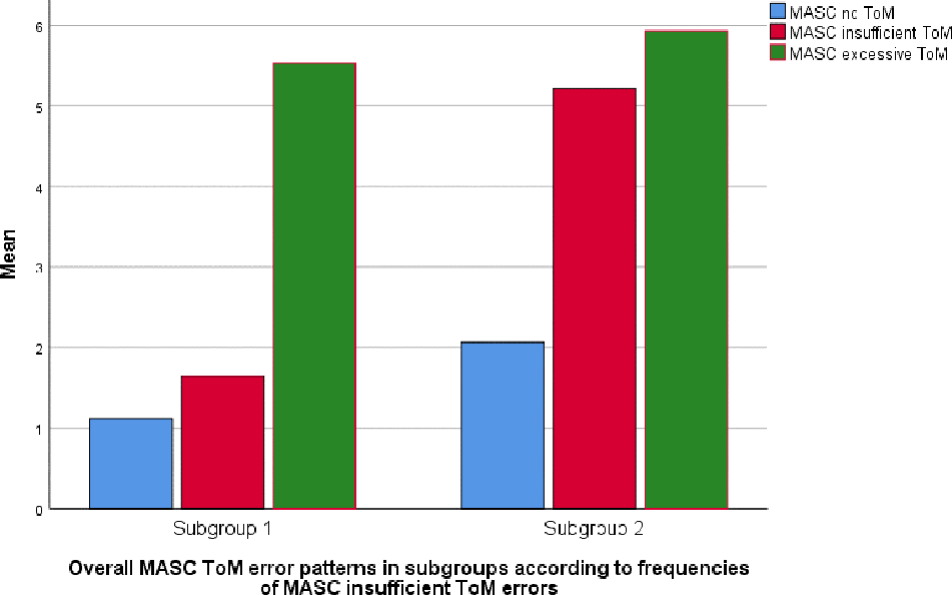
MASC ToM error patterns among BPD patients referred to MBT. **Figure 3** demonstrates ToM error profiles for patients with higher (subgroup 2) and lower (subgroup 1) levels of insufficient ToM errors according to the MASC test.

Excessive ToM errors were the dominating error type in the sample. The dichotomous variable for insufficient ToM errors divided the sample into subgroup 1 with lower error levels (55%), and subgroup 2 with higher error levels (45%). Excessive ToM errors were the main error type in subgroup 1. Subgroup 2 had a combination of error types, both excessive and insufficient, and significantly higher total levels of error types and lower levels of accurate responses (*p*_independent sample T-test_ < 0.05). **Table 3** and **Figure 3** demonstrate the distribution of MASC ToM scores in the whole sample and differences between the two subgroups. In MM analyses with total MASC errors as dependent variable, adding the dichotomous ToM variable as a predictor, subgroup 2 was significantly associated with higher levels of total ToM errors than subgroup 1 (**Table 4**).

**Table 3:** MASC ToM scores among BPD patients referred to MBT.

	Total BPDN = 31	Subgroup 1(45%)	Subgroup 2(55%)
	*Mean (SD)*	*Mean (SD)*	*Mean (SD)*
Errors, total number	10.5 (4.6)	8,3 (3,7)	13,2 (4,3)**
No ToM	1.6 (1.8)	1.1 (1.7)	2.1 (1.9)
Insufficient ToM	3.3 (2.4)	1.7 (1.3)	5.2 (1.8)***
Excessive ToM	5.7 (3.6)	5.5 (2.8)	5.9 (4.4)
Accurate responses, total number	34.7 (4.5)	36,7 (3,7)	32,1 (4,2)**
Thoughts	3.4 (0.8)	3.7 (0.6)	3.0 (0.9)*
Intentions	10.7 (1.6)	11.1 (1.7)	10.2 (1.6)
Emotions	11.1 (1.8)	11.9 (1.4)	10.1 (1.8)**

Comparison of subgroups with lower (Subgroup 1) or higher (Subgroup 2) levels of insufficient ToM errors: Independent sample T-test, ** p < 0.01, ***p < 0.001

**Table 4:** MASC ToM errors and clinical baseline status—mixed model estimations.

MM		Intercept estimate	Explained residual variation	Model fit
Model	*Predictor*	Mean (SE)	%	AIC
ToM errors, total number		10,5 (0.22)	Ref. 20.8(1.5)***	2370
	Subgroup 1	-4.9(0.38)***	*29*	2235
	Subgroup 2	*Ref*		
Insufficient ToM errors		3.26 (0.1)	Ref. 5.4(0.4)***	1824
	Avoidant PD criteria	0.38(0.08)***	*6*	1798
	PTSD	1.46(0.28)***	*7*	1737
	Sum childhood trauma	0.55(0.07)***	*13*	1761
	Sum prior treatment	0.06(0.03)*	*19*	732
	Irregular attendance	1.32(0.23)***	*13*	1715

**Table 4** demonstrates mixed model estimations (MM) for two dependent MASC variables (the total number of errors and the number of insufficient ToM errors) and associated baseline deviation (intercept estimates and % explained variation). Significant differences are marked with * (p < 0.05) or ***(p < 0.001). Indicator of model fit is Akaikes Information Criterion (AIC). The two subgroups indicate patients with lower (Subgroup 1) or higher (Subgroup 2) levels of insufficient ToM errors.

In MM models with insufficient ToM errors as dependent variable, the number of BPD criteria and avoidant PD criteria were in separate models, both associated with higher levels of insufficient ToM, as was the presence of comorbid PTSD (p_MM_ < 0.05). In models combining PTSD with avoidant and BPD traits, BPD traits were no longer significant predictors. Overall the sample displayed noteworthy risk of self-harm, suicide attempts, and aggressive behaviors, history of childhood trauma or neglect, and extensive previous treatment experience. A small proportion reported transient psychotic episodes (**Table 5**). More insufficient ToM errors were associated with having higher scores of childhood trauma, more extensive prior treatment experience and irregular treatment attendance in the current treatment (p_MM_ < 0.05). Insufficient ToM errors were not associated with treatment drop-out (p_MM_ >0.05). Suicide/self-harm, violence and total risk scores did not explain variation of insufficient ToM errors (p_MM_ >0.05). **Table 4** demonstrates the significant MM estimates including % explained variation and improvement of model fit. **Table 5** demonstrates the pretreatment distribution of risk-prone situations and treatment status.

**Table 5:** Background and treatment factors according to MASC profile.

	Subgroup 1 (N = 17)		Subgroup 2 (N = 14)	
	Mean(SD)	%	Mean(SD)	%
*Baseline risk assessments*				
Suicide attempts last year		25		23
Self-harming last year		100		93
*Suicide/Self-harm risk score*	1.63 (0.6)		1.54 (0.8)	
Physical violence (people)		18		14
Physical violence (things)		29		50
Record of police report		6		7
*Violence risk score*	0.52 (0.9)		0.71 (0.9)	
Transient psychotic episodes		13		17
*Total risk score*	2.27 (1.0)		2.45 (1.6)	
*Childhood trauma*				
Parents’ divorce < age 10 yrs		35		36
Loss of close attachment before 10 yrs		12		29
Severe trauma/illness		12		7
Sexual assault		18		36
Other physical violence		12		36
Neglect of care		42		50
Sexual abuse		12		7
*Sum childhood trauma*	1.65 (1.4)		2.00 (1.8)	
*Previous treatment*				
Age first time	14 (4)		19 (6)	
Number of treatment periods	5 (3)		4 (2)	
Psychiatric hospital admissions	2.9 (2)		4.9 (6.9)	
Medication >6 months		47		69
*Sum prior treatment score*	10.12 (1.9)		12.20 (9.5)	
*Treatment compliance MBT*				
Completed according to plan		56		50
Regular attendance		56		43
Early termination		13		7
Advised termination		31		29
Classified as drop-out		13		14
Irregular attendance		31		50
Treatment duration (years)	1.47 (0.9)		1.71 (0.9)	

**Table 5** demonstrates descriptive background and treatment data in the two subgroups, subgroup 1 with lower levels of insufficient ToM errors and subgroup 2 with higher levels.

## Discussion

The present study is a small, observational investigation. It represents an exploration of how social cognition may be associated with psychotherapeutic alliance and outcomes in MBT. Its relevance relates to the core focus of MBT. The study is limited to assessing the theory of mind concerning *other* people. In MBT, therapists will aim to focus and explore the understanding of both self and other. In essence, all psychotherapy formats represent social settings where interpersonal aspects of theory of mind are continuously shaping the dialogue, both implicitly, unconsciously, and explicitly focused. It is the aim of MBT to enhance such capacity. However, in the present study we report noteworthy differences in process and outcomes associated with patients’ pretreatment capacity for mentalizing other people.

### Main Findings

The main findings in this study are summarized in the following: In accordance with former research (17), excessive ToM errors, also termed hypermentalization, were in this study, dominating ToM errors and characteristic of the sample as a whole. However, contrary to our hypothesis, excessive ToM errors did not impede alliance development over time nor clinical improvement. In this study, insufficient ToM errors had no significant effect on alliance development, but were strongly associated with poorer clinical improvement over time.

The secondary analyses provided further exploration of impairment of social cognition within the sample, and indicated that patients with higher levels of insufficient ToM also had higher levels of excessive ToM errors and higher total error levels. Insufficient ToM errors were associated with comorbid avoidant PD traits, current PTSD, more extensive childhood trauma history, and treatment irregularity. Higher rates of violent or self-destructive behaviors were not associated with higher levels of insufficient ToM errors.

Hypermentalization indicates exaggerated, relationally hypervigilant, social misinterpretations. BPD patients may be vulnerable in close relationships, balancing a need for social attachment with easily activated fear of rejection. The problematic consequence of high sensitivity and negative bias, can be interpersonal misinterpretations, repeated conflicts, and unstable relationships (1). However, hypermentalizing problems are not always constant, and BPD patients may have far better mentalizing capacity in less dysregulated situations (8). Such variation can be seen to represent therapeutic potentials.

Contrary to our expectations, the present MBT study indicates that the extent of hypermentalizing problems did not impede long-term outcomes. In the current sample, clinical outcomes indicated an average improvement over time. Favorable improvement trends have been demonstrated in former, larger sampled studies (18, 45). As a specialized BPD treatment, MBT will address the relation self-other by work in therapy dyads, groups and direct exploration of significant interpersonal incidents, emotional reactions, and social interpretations (46). Several qualitative studies describing change processes among BPD patients have indeed highlighted the subjective impact of new relational experience, practice, and competence (47–50). Positive patient-reported group experiences—learning to gain and lend perspectives are indeed emphasized in a qualitative study of MBT (51). In our study, patient’s self-report indicated improvement of interpersonal problems among patients with mainly hypermentalizing problems. Unfortunately, we have no assessment of how the capacity for social cognition changed during treatment.

Patients’ experience of alliance may also indicate relational capacity. In our study the ratings of working alliance in the early phase of treatment, were generally within a good range. This was somewhat surprising as it was irrespective of the MASC profile. A possible explanation is that it may be easier to follow the treatment in the first phase of MBT, in the period providing more structured psychoeducation. Our study indicates that patients who tended to hypermentalize felt increasingly more able to make a bond to their therapist and find agreement on aims and tasks of therapy. Establishment of a working alliance is known as a cornerstone of psychotherapy, closely associated with outcomes (52). Establishing a good match between patient and therapist is crucial. Although hypermentalizing problems certainly represent relational challenges, these did not hinder the therapeutic process. It may be that such problems are easily identified and that the MBT approach provides strategies for addressing such situations.

A highly consistent finding of the present study is related to the MASC error type termed “insufficient theory of mind.” These are social interpretations with some reference to another person’s mind, but often missing the point or lacking relational nuance. It is conceivable that such misinterpretations will deviate sharply from the immediately appropriate situational understanding. Our data suggest a strong, but negative, clinical impact. An increasing number of insufficient theory of mind errors were clearly, and consistently, associated with poorer clinical outcomes. Although the sample is small, and findings need replication in larger samples, the result was evident across a range of outcome measures, including both patient-reports and clinician-rated. On the basis of the present data, we cannot conclude that such mentalizing deficiencies impaired development of working alliance over time. However, the overrepresentation of treatment irregularity among the patients with insufficient theory of mind errors, may nevertheless, indicate a lack of relational attachment and bonding in therapy. Social interpretation with inadequate relational reference, can on one hand, if explicit, contribute to conflictual situations or social misunderstandings. On the other hand, uncertainty about social interpretation, may also lead to less explicit behaviors, more introversion, social resignation, and even isolation. Either way, interpersonal incidents, reactions, or behaviors, are likely to also come to play in therapeutic settings, not least in therapy groups. For these patients, positively reinforcing interpersonal experiences may have been harder to achieve in therapy.

In the present study, imprecise interpretations of other peoples’ thoughts seemed the most problematic. Interpretation of emotions or intentions did not explain further variation. Although MBT aims to improve reflection over mental states, a therapist focus on emotional states or emotionally loaded situations may be a more apparent starting point for both therapists and patients. Our results suggest that poorer capacity for interpreting cognitive aspects of other people’s mind-states represent a greater treatment challenge. It could be that therapists to a lesser extent manage to identify and explore how adequately a patient perceives what other people may be thinking. Our data do not provide grounds for further understanding of personality profiles or emotionality among the patients with more impaired interpretation of cognitive mind states.

Insufficient theory of mind errors or corresponding concepts indicating poorer sensitivity to social cues, have been suggested in studies of BPD (7), but are, nevertheless, not well documented. In our study, they were not the most frequent error type within the sample. It is noteworthy that insufficient theory of mind errors were particularly associated with BPD comorbidity, not BPD features alone. The contribution of comorbid avoidant PD was significant, together with a traumatic background and current traumatic distress. In former studies, attachment anxiety, severe mentalizing problems, poor affect consciousness, impaired psychosocial functioning, and questionable treatment response have all been associated with avoidant PD (11, 53–55). Interestingly, in our study, high severity in terms of affinity for risk-prone situations did not seem characteristic of the subgroup with insufficient theory of mind errors.

Our study points to a highly vulnerable subgroup with more extensive theory of mind errors and inconsistent error patterns, becoming inaccurate both in terms of exaggerated relational interpretations (hypermentalizing) and by underestimating relational cues (insufficient mentalizing). It would thus seem that these patients represent considerable problems of social cognition. In other MASC studies, the two error domains, insufficient theory of mind and no theory of mind (concrete, non-relational, non-mentalistic explanations of social interactions) have been combined as a variable indicating “undermentalizing” (56). In our study, no theory of mind error types were infrequent, and in our data analyses such a combined variable did not explain additional variation. In the present sample no patients had received a clinical diagnosis within autism spectrum, but our study did not include dimensional information concerning a more moderate degree of such problems. However, these conditions can be hard to distinguish. A recent qualitative study of avoidant PD suggests impairment of tacit knowledge of social behavior (57).

### Strengths and Limitations

The recruited sample consisted of clinically severe BPD patients referred on a regular basis to an ongoing treatment service on a specialist mental health service level. It is a cohort which may differ from more selected BPD research samples or studies recruiting from nonclinical cohorts. Pretreatment diagnostic assessments also confirm that the patient-intake to the MBT program, followed the current recommendations for MBT—poorly functioning patients with BPD and severe disorder. It is a strength that the sample represents a treatment seeking BPD cohort.

The applied test for social cognition (MASC) is a well-established, validated method used across several psychiatric populations and translated versions (8, 16, 56). However, a limitation is nevertheless that the MASC test implied that a dubbed movie was presented for participants. The movie quality may effect a more finely attuned interpretation of social interactions.

It is a strength that this MBT study provides documentation of treatment fidelity. As yet, few MBT studies have reported on treatment fidelity, and seldom both the group and individual therapy components of the treatment. The MBT fidelity measures are based on the manuals used by the therapists and their reliability has been tested and reported (39, 40). In the present study, overall levels of MBT quality with respect to therapists’ in-session interventions were satisfactory, indicating reasonable model adherence. However, we do not have session by session quality assessments over time, nor assessments of all therapies. There is reason to expect that the quality in this respect may be variable, in particular when levels of mentalizing are poor and/or the situations are highly emotional or indicative of high-risk such as violent or self-destructive behaviors (58). Moreover, the data does not allow distinction between treatment fidelity in dyads were the patient had more impaired social cognition.

The study has a longitudinal design enabling explorative investigation of change of alliance and change of functioning, symptoms, and interpersonal problems over time. Assessments and diagnostic evaluations were based on validated instruments, both observer-rated and self-report. As a study based on regular clinical practice, assessment procedures held a systematic, high standard. However, inter-rater reliability of diagnostic interviews were not performed.

The main limitation of this study is the small sample size. However, we nevertheless, choose to present the results as there are, currently, few investigations with this focus. We consider our findings of sufficient interest to recommend further investigation and replication in larger samples. The longitudinal data are also unbalanced with uneven numbers at each assessment. To compensate for unbalanced data, advanced longitudinal statistics were applied based on maximum likelihood statistics with individual trajectories. We have also included analyses investigating the possible bias of different numbers of assessment. The study design is observational, exploring within sample variation. The investigation can indicate associations between variables, but cannot answer questions of causality.

## Conclusion

The study demonstrates variation in capacity for social cognition among poorly functioning BPD patients admitted to a specialized treatment. While it suggests good outcomes for BPD patients with mainly ToM errors of hypermentalizing, it also indicates that poorly responding patients may represent a cohort with more complex problems of social cognition and comorbidity. The study casts light on the heterogeneity within a clinical cohort of BPD patients, possibly implicating a need for individualized treatment strategies within specialized frameworks.

## Data Availability Statement

The datasets generated for this study are available on request to the corresponding author.

## Ethics Statement

The studies involving human participants were reviewed and approved by Regional Committee for Medical and Health Research Ethics, Norway. The patients/participants provided their written informed consent to participate in this study.

## Author Contributions

The author’s contributions to this manuscript are as follows: Data-collection, statistical analyses, presentation of results, main responsibility for the manuscript (EK), MBT fidelity assessments (EF), MASC tests (BA), data-collection (EN-E), research database and psychometric evaluations (GP), principle investigator (TW). All authors have contributed in discussion of MASC tests, results, and development of the manuscript.

## Conflict of Interest

The authors declare that the research was conducted in the absence of any commercial or financial relationships that could be construed as a potential conflict of interest.
